# Effect of Vitamin E and Omega-3 Fatty Acids on Protecting Ambient PM_2.5_-Induced Inflammatory Response and Oxidative Stress in Vascular Endothelial Cells

**DOI:** 10.1371/journal.pone.0152216

**Published:** 2016-03-23

**Authors:** Liang Bo, Shuo Jiang, Yuquan Xie, Haidong Kan, Weimin Song, Jinzhuo Zhao

**Affiliations:** 1 Department of Environmental Health, School of Public Health and the Key Laboratory of Public Health Safety, Fudan University, Shanghai 200032, China; 2 Department of Cardiology, Xinhua Hospital, Shanghai Jiao Tong University, School of Medicine, Shanghai, 200092 China; University of Chinese Academy of Sciences, CHINA

## Abstract

Although the mechanisms linking cardiopulmonary diseases to ambient fine particles (PM_2.5_) are still unclear, inflammation and oxidative stress play important roles in PM_2.5_-induced injury. It is well known that inflammation and oxidative stress could be restricted by vitamin E (Ve) or omega-3 fatty acids (Ω-3 FA) consumption. This study investigated the effects of Ve and Ω-3 FA on PM_2.5_-induced inflammation and oxidative stress in vascular endothelial cells. The underlying mechanisms linking PM_2.5_ to vascular endothelial injury were also explored. Human umbilical vein endothelial cells (HUVECs) were treated with 50 μg/mL PM_2.5_ in the presence or absence of different concentrations of Ve and Ω-3 FA. The inflammatory cytokines and oxidative stress markers were determined. The results showed that Ve induced a significant decrease in PM_2.5_-induced inflammation and oxidative stress. Malondialdehyde (MDA) in supernatant and reactive oxygen species (ROS) in cytoplasm decreased by Ve, while the superoxide dismutase (SOD) activity elevated. The inflammatory cytokines interleukin 6 (IL-6) and tumor necrosis factor α (TNF-α) also reduced by Ve. Moreover, Ω-3 FA played the same role on decreasing the inflammation and oxidative stress. IL-6 and TNF-α expressions were significantly lower in combined Ve with Ω-3 FA than treatment with Ve or Ω-3 FA alone. The Ve and Ω-3 FA intervention might abolish the PM_2.5_-induced oxidative stress and inflammation in vascular endothelial cells. There might be an additive effect of these two nutrients in mediating the PM_2.5_-induced injury in vascular endothelial cells. The results suggested that inflammation and oxidative stress might be parts of the mechanisms linking PM_2.5_ to vascular endothelial injury.

## Introduction

Particulate matter has been the main air pollutant threatening human health in the world. The particles with aerodynamic diameters less than 2.5 μm (fine particles, PM_2.5_) have been regarded as the most detrimental air pollutant. In autumn and winter of the latest three years in China, the haze lay over the whole Shanghai and Beijing, and the concentration of PM_2.5_ even exceeded 600 μg/m^3^. The association between PM_2.5_ and cardiopulmonary diseases [1], cerebrovascular disease [2], and diabetes [3] has been comprehensive studied in epidemiological and experimental studies. Although the mechanism linking PM_2.5_ and cardiopulmonary diseases is unclear, the previous studies have demonstrated that the inflammatory response and oxidative stress may be responsible [4,5]. Oxidative stress can occur through air pollutant-induced reactive oxygen species (ROS) production with decreased antioxidant enzyme activity. The cardiovascular events including ischemic heart disease and myocardial infarction, are associated with the vascular endothelial dysfunction, and increased circulating levels of inflammatory cytokines, such as interleukin 6 (IL-6) and tumor necrosis factor α (TNF-α), which may be raised by the modifiable environmental factors [6].

With adverse effect of the high pollution of PM_2.5_, the government and researchers have been attempting to find ways to protect people’s health. Sufficient nutrition may contribute to protect individuals against the external pathogens. Antioxidants and anti-inflammatory agents have been evaluated as strategies to block the respiratory effects induced by ambient air pollution exposure [7]. Vitamin E (Ve) and omega-3 fatty acids (Ω-3 FA), the important nutrients in maintaining people’s health, have been verified to be the antioxidants and anti-inflammatory agents [8,9,10]. Ve supplementation was effective on blunting ozone-induced decrements in lung functions in healthy adults [11]. Therefore, Ω-3 FA and Ve might play a vital role in alleviating the air pollution-induced injury. We hypothesized that Ve and Ω-3 FA could influence the cardiovascular injury induced by PM_2.5_, and combination of Ve and Ω-3 FA may have additive effect. This study was conducted to explore the potential mechanisms linking PM_2.5_ and vascular endothelial function, and to investigate the combined effect of these two nutrients.

As the wall of blood vessels and the blood stream, the pathophysiology of endothelium is complex and involves multiple mechanisms. Endothelial dysfunction has been proposed to be a key risk factor in the development of cardiovascular diseases [12]. Meanwhile, endothelial dysfunction can promote inflammation and platelet adhesion that might aggravate the cardiovascular injury. Previous study demonstrated that systemic endothelial dysfunction was induced when exposed to ambient particulate matter by instillation [13]. Our previous study also indicated that ambient PM_2.5_ was associated with the inflammatory response and vascular endothelial dysfunction in human umbilical vein endothelial cells (HUVECs) [14].

In this study, HUVECs were treated with ambient PM_2.5_ and nutrients to determine whether inflammation and oxidative stress caused by ambient PM_2.5_ could be blunted by Ve or Ω-3 FA acid. This study also attempted to find the appropriate dosage for combination of Ve and Ω-3 FA in preventing the PM_2.5_-induced HUVECs injury.

## Materials and Methods

### Cell culture

The HUVECs were cultured in RPMI1640 medium supplemented with 10% fetal bovine serum. The HUVECs were cultured at 37°C in humidified air containing 5% CO_2_. The cells were treated with PM_2.5_ in the presence or absence of different concentrations of nutrients for 24 h. Then biomarkers in the cells and supernatant were detected.

### PM_2.5_ preparation

The ambient PM_2.5_ were captured on the roof of a building in downtown Shanghai (10 meters high or so, without obvious source of pollution around), by Thermo Anderson G-2.5 with large-volume sampler glass fiber filters.

The dry particles was formulated in PBS at a concentration of 1000 μg/ml and stored at 4°C. The suspension was diluted in culture medium to the final working concentrations for this study.

### Nutrients preparation

Both Ve and Ω-3 FA were provided by Royal DSM Company (Ve from Kaiseraugst, Switzerland; Ω-3 FA from Halifax, Canada) and diluted into complete RPMI1640 medium to get the initial working concentrations and stored at -4°C. The solution was sonicated before the experiment.

### Experimental design

The treatment groups were divided into 24 groups. For all the blank groups and treatment groups, the HUVECs were treated with 0 μg/mL (control) or 50 μg/mL (PM_2.5_). The detailed groups were as follows:

*Blank groups*:The HUVECs were treated with medium or PM_2.5_ only.
*Ve treatment groups*: The HUVECs were treated with medium or PM_2.5_. Simultaneously, the cells were treated with 10 μmol/L, 20 μmol/L, or 50 μmol/L of Ve, respectively.
*Ω-3 FA treatment groups*: The HUVECs were treated with medium or PM_2.5_. Simultaneously, the cells were treated with 6 mg/L, 12 mg/L or 30 mg/L of Ω-3 FA, respectively.
*Combined action groups*: After treated with medium or PM_2.5_, the HUVECs in low-dose group were treated with 10 μmol/L of Ve and 6 mg/L of Ω-3 FA; The HUVECs in medium-dose group were treated with 20 μmol/L of Ve and 12 mg/L of Ω-3 FA; The HUVECs in high-dose group were treated with 50 μmol/L of Ve and 30 mg/L of Ω-3 FA. The HUVECs in recommended-dose group were treated with 3 μmol/L of Ve and 30 mg/L of Ω-3 FA.

After treatment with PM_2.5_ and nutrients, the cell viability, inflammation and oxidative stress were determined in cells. The quantitative measurements of all the experiments and endothelial cell activation markers were performed in triplicates.

### Cell viability determination

The cytotoxicity was determined by MTT-based experiment. Results were expressed as spectrometric absorbance values at 570 nm.

### Cell membrane integrity assessment

Lactate dehydrogenase (LDH), which expresses in the cytoplasm, plays an important role in the life of the body energy metabolism (mostly glycolysis). When the cell membrane is damaged, the intracellular LDH will be released from cells. The cell permeability can be measured by supernatant LDH/total LDH (supernatant LDH/ supernatant LDH + intracellular LDH).

The cell culture supernatant was collected for further assessment before the cells were treated with 0.1% Triton X-100 to acquire cell lysate. Both supernatant and lysate were used to detect the level of LDH. Then the supernatant LDH/total LDH was calculated.

### Intracellular oxidative stress determination

Malondialdehyde (MDA) is the production of lipid peroxidation of cell membrane, which can reflect the severity of oxidative stress. In this study, supernatants MDA were detected by the thiobarbituric acid (TBA) reaction method. Superoxide dismutase (SOD) is an antioxidant intracellular enzyme, which is a sensitive indicator of cell antioxidant capacity. SOD was determined by the xanthine oxidase test. ROS was detected with probe 2,7-dichlorodihydrofluorescein diacetat (DCFH-DA, Sigma). Probe DCFH-DA penetrates the cells and is hydrolyzed by intracellular esterases to the nonfluor- escent DCFH, which can be rapidly oxidized to the highly fluorescent 2,7-dichlorofluorescein (DCF) in the presence of ROS. Relative ROS production was determined by the formation of a DCF compound [15]. After cells were stimulated with 50 mg/ml PM_2.5_ suspension and nutrients for 24 h, the cells were incubated with 10 mM DCF-DA at 37°C for 30 min and washed twice with PBS. The fluorescence microscope (Olympus BX51, Japan) was used to take the pictures for observing the qualitative expression of ROS. Relative fluorescence was measured using a fluorescence plate reader at 485 nm excitation and 535 nm emission wavelength (Hitachi, Japan).

### Inflammatory cytokines determination

The levels of IL-6 and TNF-α in HUVECs were quantified by enzyme-linked immunosorbent assay (ELISA).

### Statistics analysis

The results are expressed as Mean±SD for different experiments, and each assay was performed in triplicates (n = 3). Statistical significance was calculated by one-way analysis of variance (ANOVA). Multiple comparisons were decided by LSD-*t* test or Dunnett's test, and *p* values of < 0.05 are considered significant. Error bars represent standard deviation of the mean. All data were analyzed by SPSS version 16.0.

## Results

### Cell viability and cell membrane integrity

**Fig 1:**
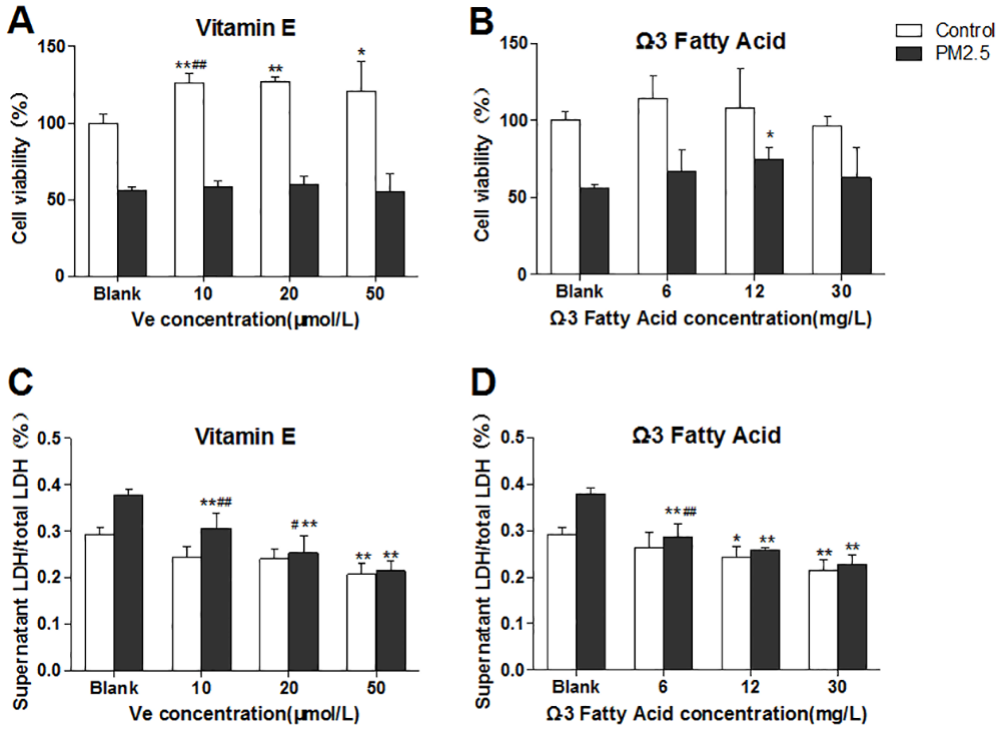
Cell viability & membrane integrity in HUVECs after treated with vitamin E (Ve) and Ω-3 fatty acids (Ω-3 FA) (n = 3 per group). The blank group has no nutrients. The control groups have no PM_2.5_. A&B, representative cell viability; C&D, membrane integrity. Significant difference **p< 0.01, *p< 0.05 vs. blank group; ^#^*p* < 0.05, ^##^*p* < 0.05 vs. previous group in the bar graph.

As shown in Fig 1, Ve treatment induced the increase of cell viability in control group but not in PM_2.5_ treatment groups. In Ω-3 FA-treated group, the cell viabilities have no significant changes except the medium-dose PM_2.5_-treated group. (Fig 1A & 1B).

As shown in Fig 1C, in the Ve treatment groups with PM_2.5_ existing, supernatant LDH/total LDH significantly decreased with the increase of Ve, which indicating that Ve could protect the cell membrane integrity induced by PM_2.5_. For the Ω-3 FA-treated groups (Fig 1D), supernatant LDH/total LDH exhibited distinctive dose-dependent decrease in cells when treated with PM_2.5_.

### Intracellular oxidative stress in HUVECs

**Fig 2:**
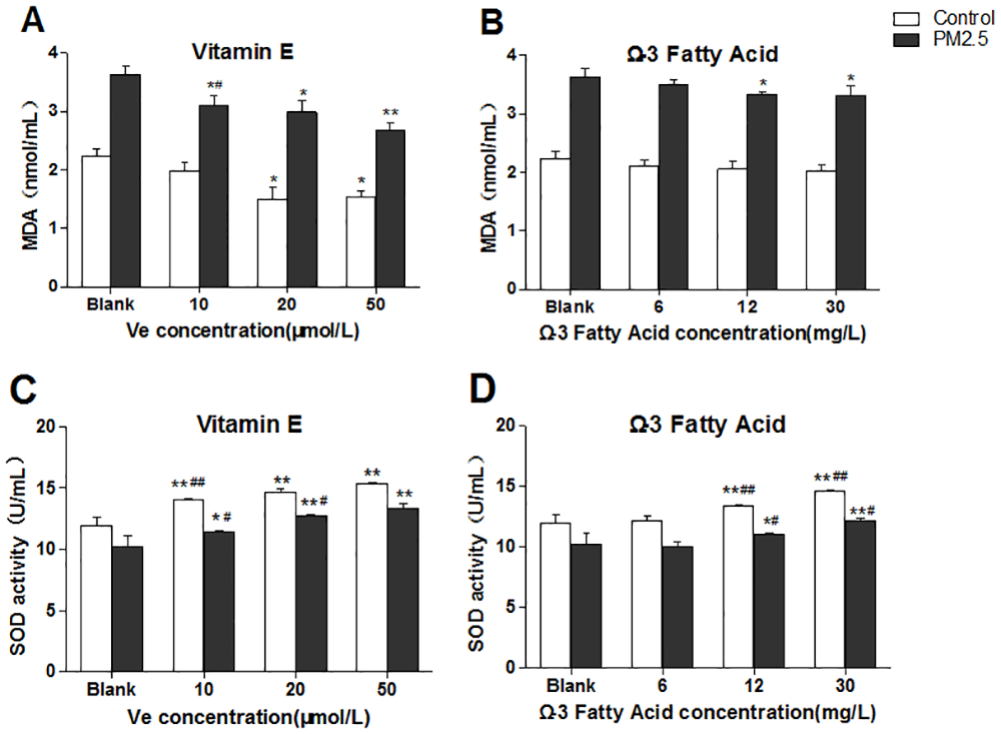
Oxidative stress in HUVECs after intervened by vitamin E (Ve) and Ω-3 fatty acids (Ω-3 FA) (n = 3 per group). The blank group has no nutrients. The control groups have no PM_2.5_. A&B, representative MDA detection of supernatant; C&D, intracellular SOD activity. Significant difference **p< 0.01, *p< 0.05 vs. blank group; ^#^*p* < 0.05, ^##^*p* < 0.05 vs. previous group in the bar graph.

To determine the oxidative stress in HUVECs, supernatant MDA, intracellular SOD activity and ROS were detected. The Fig 2A illustrated that the level of MDA significantly decreased in cells with the increase of Ve. Compared with the controls, the decrease trend of MDA induced by PM_2.5_ appeared to be more significant in the PM_2.5_ treatment group. When the concentration of Ve reaches to 20 μmol/L, the MDA starts a significant decrease. Curiously, the cells appeared to be less sensitive to Ω-3 FA, which did not induce a statistically significant reduction on MDA production. Ω-3 FA only led to a slight decrease in medium and high dose group when PM_2.5_ existing but has no dose-dependent manner (Fig 2B). A dose-dependent increase of cytoplasm SOD activity was observed in Ve or Ω-3 FA groups no matter with or without the presence of PM_2.5_ (although 6 mg/L Ω-3 FA group showed no significant changes in SOD) (Fig 2C & 2D).

**Fig 3:**
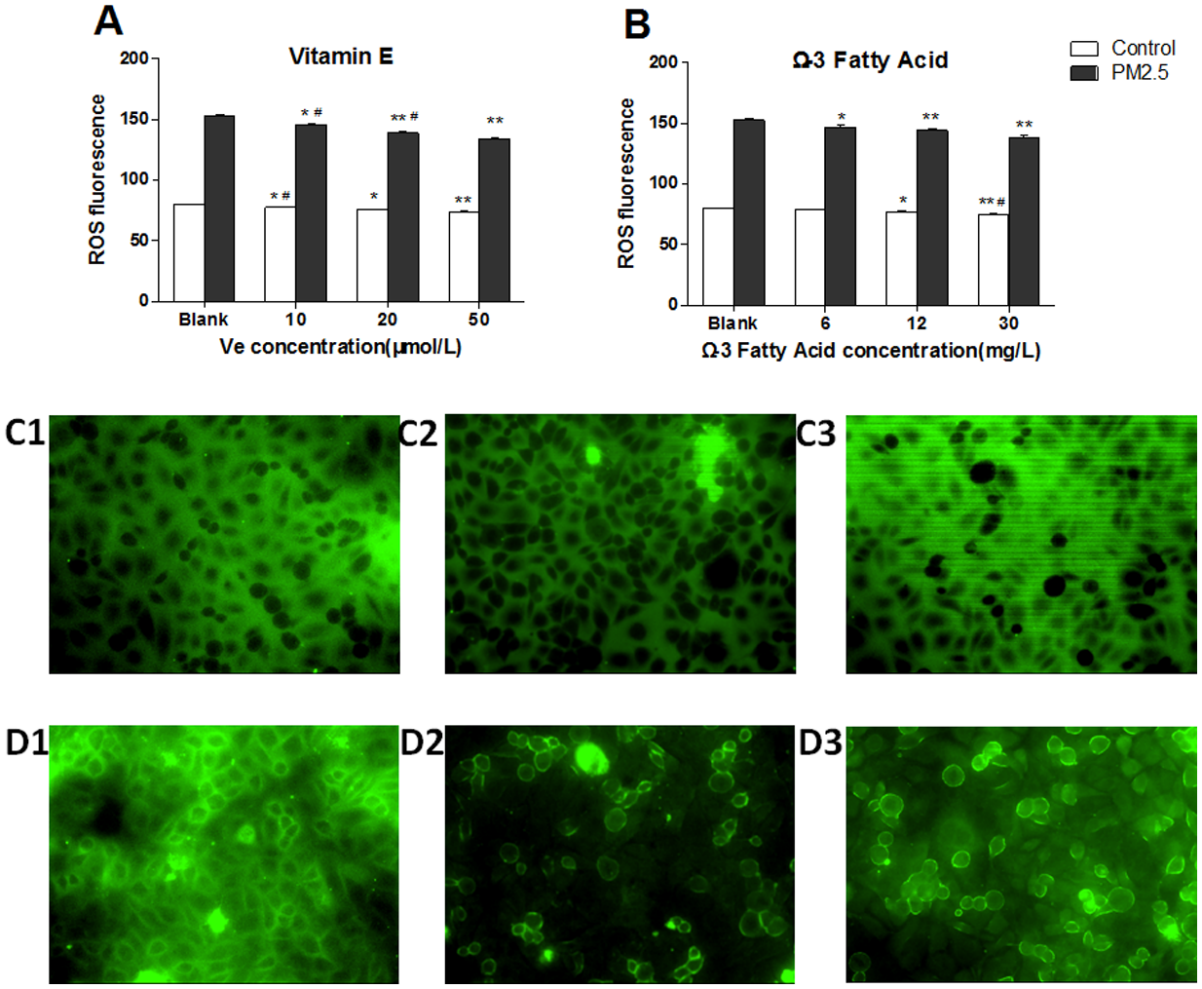
The ROS expression in HUVECs after intervened by vitamin E (Ve) and Ω-3 fatty acids (Ω-3 FA) (n = 3 per group). The blank group has no nutrients. The control groups have no PM_2.5_. A&B, representative quantitative analysis. C&D, the confocal laser scanning microscope images of cells (200× in all groups); C1–C3 represented control groups; D1–D3 represented PM_2.5_ groups; From1-3, it represented the exposed dose of nutrients, blank, Ve 50 μmol/L, Ω-3 FA 30mg/L. Significant difference **p< 0.01, *p< 0.05 vs. blank group; ^#^*p* < 0.05, ^##^*p* < 0.05 vs. previous group in the bar graph.

The expression of intracellular ROS was detected in cells. According to the Fig 3, the ROS level illustrated a dose-dependent decline on both Ve and Ω-3 FA treatment groups, and the level of ROS in PM_2.5_ groups exhibited a significant decrement when compared with the control groups. Meanwhile, the ROS qualitative determination using fluorescence microscope also provided the similar findings (Fig 3C and 3D).

### The levels of inflammatory cytokines

**Fig 4:**
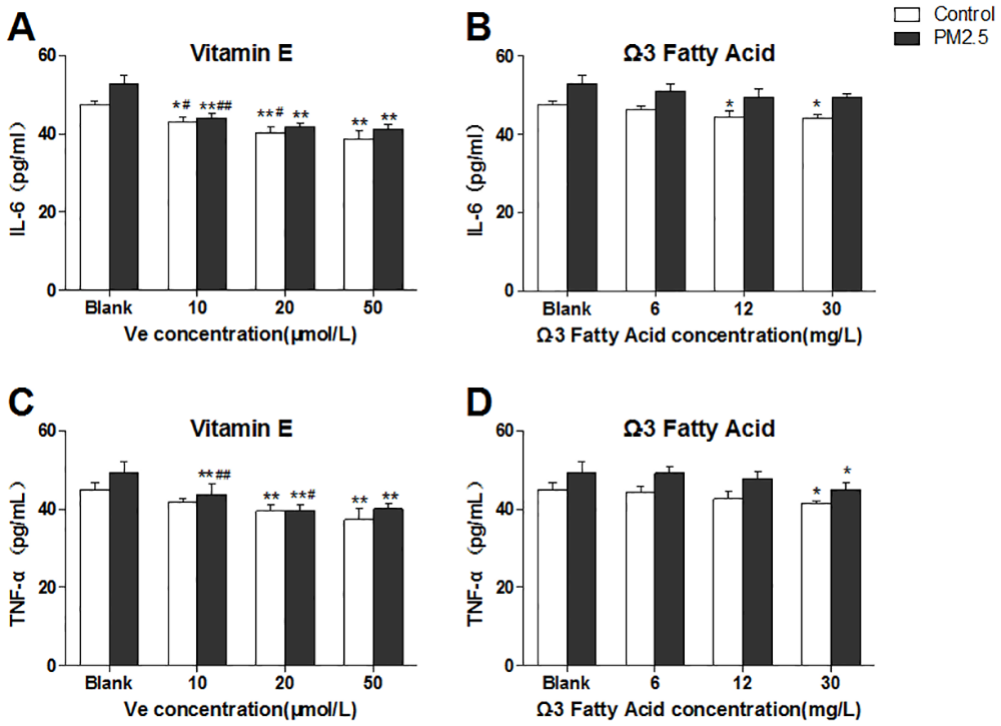
Inflammatory cytokines expression in HUVECs after intervened by vitamin E (Ve) and Ω-3 fatty acids (Ω-3 FA) (n = 3 per group). The blank group has no nutrients. The control groups have no PM_2.5_. A&B, representative IL-6 detection; C&D, TNF-α detection. Significant difference **p< 0.01, *p< 0.05 vs. blank group; ^#^*p* < 0.05, ^##^*p* < 0.05 vs. previous group in the bar graph.

As shown in Fig 4, Ve induced a dose-dependent decline of IL-6 and TNF-α in both control and PM_2.5_ groups (Fig 4A & 4C). However, there were no dose-dependent changes on the production of IL-6 in the Ω-3 FA-treated cells when there is the presence of PM_2.5_ (Fig 4B). Similarly, compared with the blank groups, the level of TNF-α also showed no significant changes in nutrient-treated groups excluding the high-dose Ω-3 FA-treated groups (Fig 4D).

### Effects of combined action

**Fig 5:**
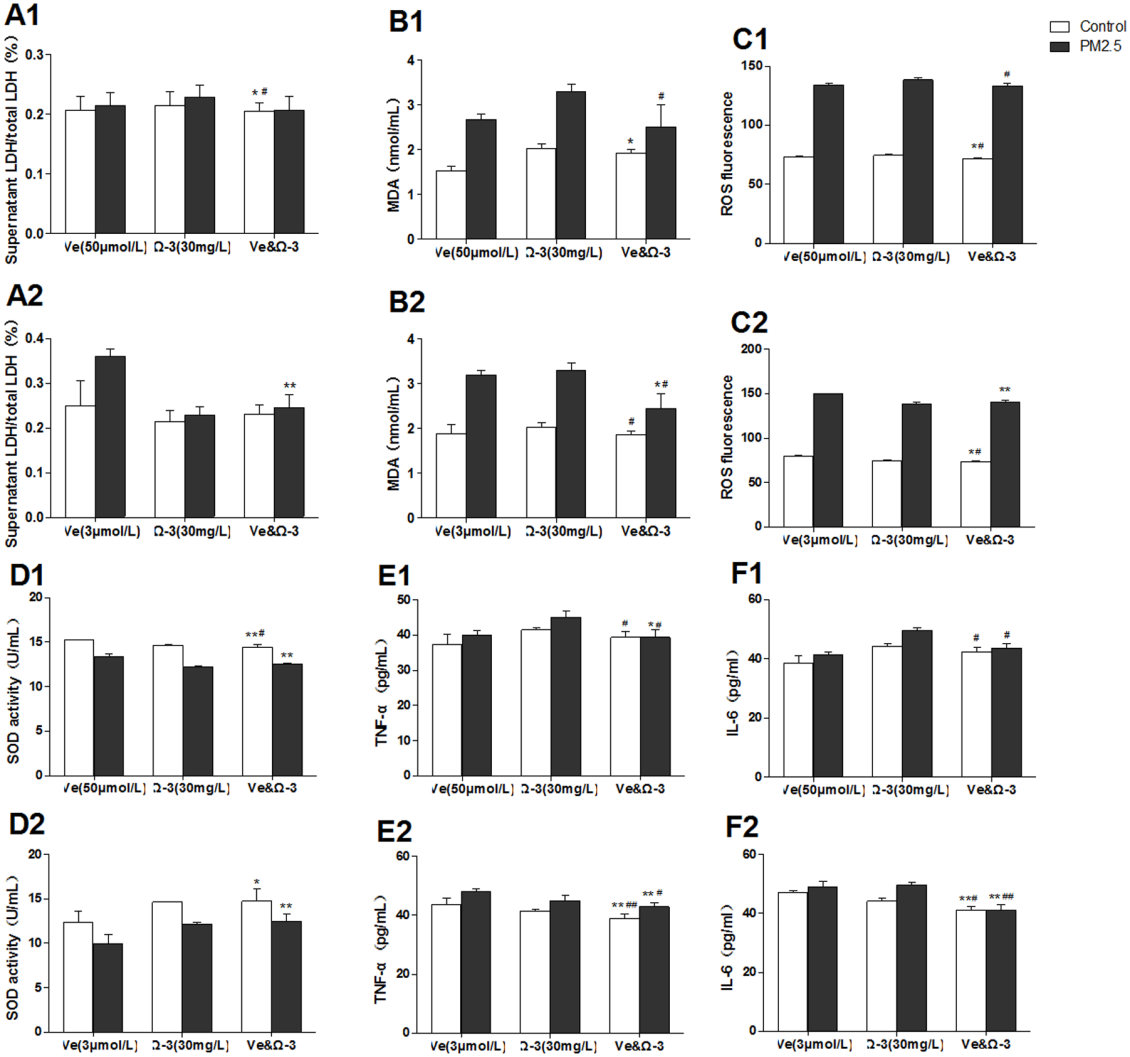
Combined action of vitamin E (Ve) and Ω-3 fatty acids (Ω-3 FA) (n = 3 per group). The blank group has no nutrients. The control groups have no PM_2.5_. A1&A2, representative cell membrane integrity; B1-D2, oxidative stress of HUVECs; E1-F2, the level of inflammatory cytokines. Significant difference **p< 0.01, *p< 0.05 vs. blank group; ^#^*p* < 0.05, ^##^*p* < 0.05 vs. previous group in the bar graph.

In order to find appropriate combined doses of Ve and Ω-3 FA in preventing PM_2.5_-induced injury, different proportions of Ve and Ω-3 FA were set to protect the PM_2.5_-treated cells according to our previous results. The doses included Ve (50 μmol/L)/Ω-3 FA (30 mg/L) or Ve (3 μmol/L)/Ω-3 FA (30 mg/L). The Fig 5A1 & 5A2 illustrated that there was no significant combined action on cell membrane integrity. The oxidative stress assays showed that the combination of Ve and Ω-3 FA induced the decrease of MDA and ROS in HUVECs than the separated ones (Fig 5B1, 5B2, 5C1 & 5C2). In addition, the combination of Ve and Ω-3 FA also improved the SOD activity although there was no statistically alteration (Fig 5D1 & 5D2).

Nevertheless, the inflammatory cytokines determination showed a distinctive result. The production of IL-6 and TNF-α in cells treated with combined nutrients were markedly less than those in the cells treated with separate nutrient. Especially, the combined action in Ve (3 μmol/L)/ Ω-3 FA (30 mg/L) group is greater in inducing the reduction of inflammation (Fig 5E1, 5E2, 5F1 and 5F2).

## Discussion

Air pollutants, especially ambient PM_2.5_, have become important risk factor in the development of cardiovascular diseases. Epidemiological survey has confirmed an association between PM_2.5_ and increased risk of cardiovascular morbidity and mortality [16]. Recent study has reported that ambient PM_2.5_ exposures independently induced acute systemic inflammatory responses [17]. Exposure to concentrated airborne particles increases the steady-state concentration of ROS in the rat’s lung and heart with decrement on the activities of the antioxidant enzymes superoxide dismutase and catalase [18], which seems to be the most likely mechanisms in cardiovascular injury caused by PM_2.5_ [6,19,20]. Previous studies have found that dietary Ve might improve antioxidant metabolism and reduce lipid peroxidation [6,21,22]. Meanwhile, the capability of Ω-3 FA inhibiting inflammation through G protein-coupled receptor 120 activation has also been reported [23]. High level of marine-derived Ω-3 FA has been proved to contribute to lower the burden of atherosclerosis in Japanese [24]. Similarly, Ve could be beneficial to aged diabetic rats by reducing free radical production [21]. However, their protective function against air pollutants has never been clarified. Endothelial cells act as gatekeepers to control the infiltration of blood proteins and circulating cells into the vessel wall and the underlying tissues [25]. Considering that the cardiovascular disorders could be caused by ambient PM_2.5_, we conducted the relative cardiovascular toxicological research *in vitro* to investigate whether Ve and Ω-3 FA consumption could decrease the vascular endothelial cells injury, thereby in turn to protect people from such hazard.

In order to observe the unknown cytotoxicity of Ve and Ω-3 FA on cells, the MTT assay was conducted to determine the cell viability. The results indicated that Ve and Ω-3 FA in our experimental doses appeared to be nontoxic to HUVECs. On the contrary, they exhibited a slightly promotion to the cell proliferation. It has been proved that cell membrane integrity is impaired after PM_2.5_ inhalation, which allows more particles reach the cytoplasm, leading to severe intracellular oxidative damage and inflammation [26]. In this study, the results demonstrated that the nutrients have protective effects on PM_2.5_-induced membrane integrity impairment.

Oxidative stress is a biochemical imbalance in which production of ROS exceeds the natural antioxidant capacity [27]. MDA can be excessively generated after ROS mediated lipid peroxidation, which in turn results in the MDA-LDL adducts production and cell membranes damage. Humans have evolved various enzymatic defenses against ROS, among which the most specific one is SOD. SOD plays a critical role in inhibiting the oxidative stress [28]. SOD has been proved to be a critical cellular defense for coping with ROS [29]. Cellular SOD can reduce O^2-^ to H_2_O_2_, then catalase reduces H_2_O_2_ to water and molecular oxygen in order to minimize oxidative damage [27]. Exposure to ambient particles leads to an imbalance of pro-oxidants and antioxidants in the cellular environment, which in turn results in the occurrence of oxidative stress. Therefore, the MDA, ROS and SOD activity were determined in this study. We found that both Ve and Ω-3 FA were able to restrict the oxidative stress caused by PM_2.5_. Ve-treated cells demonstrated significant antioxidant capacity when compared with blank groups even at lowest concentration of 10 μmol/L. Meanwhile, Ω-3 FA also exhibited significant anti-oxidative effects, especially induced the elevation in SOD activity. This study suggested that augmented ROS and MDA levels, as well as reduced antioxidant enzymes SOD activities were consistently relieved by Ve or Ω-3 FA treatment. Inflammatory response is regarded as a potential biological mechanism on PM_2.5_ induced cardiovascular injury [6]. IL-6 is a cytokine that stimulates neutrophil production and the proliferation of B-lymphocytes, and TNF-α could induce loss of endothelial barrier function and increase permeability of cell monolayers [17,30,31]. In this study, remarkable decrement in IL-6 and TNF-α was found in cells when intervened with 10 μmol/L Ve, whereas there were no significant changes in Ω-3 FA treatment groups. The current results indicated that Ω-3 FA became effective just at the high-dose group (30 mg/L). The reason for this might be that the concentration is too low to be effective. Dhalla et. al.[32] reported that oxidative stress may play a central role in air pollution-induced respiratory and cardiovascular injury through its immunomodulating effects and its ability to initiate the inflammatory process, which might be the possible mechanism for Ve blunting the inflammation caused by PM_2.5_.

Except for the effect of Ve or Ω-3 FA alone, the effect of combination of these two nutrients was also evaluated in this study. Interestingly, the results indicated that different proportions of these two substances lead to different consequences. The Ve (3 μmol/L)/Ω-3 FA (30 mg/L) group appeared to be more efficient than other combination group. It might provide a candidate strategy to reinforce the productive effect of Ve and Ω-3 FA.

Our study was limited by the intervention modes of nutrients, which was supposed not to be a completely representative of the food consumption for human. Although the dosage of nutrients was strictly calculated according to human food intake and absorption rates, it still cannot totally represent the actual dietary nutrients consumption. Despite the limitation, our study included a carefully standardized experimental condition. We hypothesized that the dietary supplementation of Ve and Ω-3 FA might provide protective effects on PM_2.5_-induced vascular endothelial cells injury. These results indicated that the appropriate dosage of nutrients might contribute to prevent the PM_2.5_-induced cardiovascular injury. Meanwhile, these results also hinted that PM_2.5_-induced vascular endothelial dysfunction might be associated with the inflammatory response and oxidative stress.

## Conclusion

In summary, our data suggested that Ve and Ω-3 FA may blunt the inflammation and oxidative stress caused by ambient PM_2.5_. There is an additive effect of these two nutrients, while the intensity of combined effects depends on the different proportion of these nutrients. PM_2.5_-induced vascular endothelial dysfunction might be associated with the inflammatory response and oxidative stress.
